# Amsterdam wrist rules: A clinical decision aid

**DOI:** 10.1186/1471-2474-12-238

**Published:** 2011-10-17

**Authors:** Abdelali Bentohami, Monique MJ Walenkamp, Annelie Slaar, M Suzan H Beerekamp, Joris AH de Groot, Eva M Verhoog, L Cara Jager, Mario Maas, Taco S Bijlsma, Bart A van Dijkman, Niels WL Schep, J C Goslings

**Affiliations:** 1 Trauma Unit, Department of Surgery, Academic Medical Center, University of Amsterdam, P.O. Box 22660, 1100 DD Amsterdam, The Netherlands; 2 Department of Clinical Epidemiology, University Medical Center, Utrecht, Huispost Str. 6.131, P.O. BOX 85500, 3508 GA Utrecht The Netherlands; 3 Department of Emergency Medicine, Academic Medical Center, University of Amsterdam, P.O. Box 22660, 1100 DD Amsterdam, The Netherlands; 4 Department of Radiology, Academic Medical Center, University of Amsterdam, P.O. Box 22660, 1100 DD Amsterdam, The Netherlands; 5 Department of Surgery-Traumatology, Spaarne Hospital, Spaarnepoort 1, 2134 TM Hoofddorp, The Netherlands; 6 Department of Surgery-Traumatology, Flevo Hospital, Hospitaalweg 1, 1315 RA Almere, The Netherlands

**Keywords:** Clinical Decision Rule, Decision Rule, Diagnosis, Distal Radius Fractures, Radiography, Surgery, Trauma Surgery, Validation Study, Wrist Injury

## Abstract

**Background:**

Acute trauma of the wrist is one of the most frequent reasons for visiting the Emergency Department. These patients are routinely referred for radiological examination. Most X-rays however, do not reveal any fractures. A clinical decision rule determining the need for X-rays in patients with acute wrist trauma may help to percolate and select patients with fractures.

**Methods/Design:**

This study will be a multi-center observational diagnostic study in which the data will be collected cross-sectionally. The study population will consist of all consecutive adult patients (≥18 years) presenting with acute wrist trauma at the Emergency Department in the participating hospitals.

This research comprises two components: one study will be conducted to determine which clinical parameters are predictive for the presence of a distal radius fracture in adult patients presenting to the Emergency Department following acute wrist trauma. These clinical parameters are defined by trauma-mechanism, physical examination, and functional testing. This data will be collected in two of the three participating hospitals and will be assessed by using logistic regression modelling to estimate the regression coefficients after which a reduced model will be created by means of a log likelihood ratio test. The accuracy of the model will be estimated by a goodness of fit test and an ROC curve. The final model will be validated internally through bootstrapping and by shrinking it, an adjusted model will be generated.

In the second component of this study, the developed prediction model will be validated in a new dataset consisting of a population of patients from the third hospital. If necessary, the model will be calibrated using the data from the validation study.

**Discussion:**

Wrist trauma is frequently encountered at the Emergency Department. However, to this date, no decision rule regarding this type of trauma has been created. Ideally, radiographs are obtained of all patients entering one of the participating hospitals with trauma to the wrist. However, this is ethically and logistically not feasible and one could argue that patients, for whom no radiography is required according to their physician, are not suspected of having a distal radius fracture and thus are not part of the domain.

**Trial registration:**

This study is registered at the Netherlands Trial Register (NTR 2544) and was granted permission by the Medical Ethical Committee of the Academic Medical Center Amsterdam on 06-01-2011.

## Background

An acute trauma of the wrist is one of the most frequent reasons for visiting the Emergency Department, and fractures of the distal radius account for an estimated 17% of all fractures diagnosed [1,2]. The incidence of distal radius fractures was recently reported by Swedish researchers at an incidence of 26% per 10,000 person-years [3]. There are indications that in patients with wrist trauma most radiological examinations performed are redundant, resulting in unnecessary radiation exposure for the patient, as well as increased waiting times and a waste of resources [4]. The percentage of radiographs which reveal a distal radius fracture has been determined in a separate retrospective study conducted by our research group in the three Dutch hospitals that participate in the current study. In 2010, seventeen hundred and forty-two patients who were suspected of having a fracture were sent for X-ray series of the distal radius or carpus. Of all X-ray series which were performed on these patients, 50% did not show a fracture.

A clinical guideline concerning the X-ray referral policy may help to select and percolate patients with fractures. In 1987, Gleadhill et al. already concluded that: Clinical guidelines on selecting patients for radiography for certain injuries and emergencies reduced the overall X ray referral rate by 18,5% [5].

In 1991, Stiell et al. conducted a study which showed that the vast majority of ankle trauma patients was radiographically examined upon presentation [4]. They established that only 4.3% of these patients were found to have significant midfoot fractures and 9.3% were diagnosed with significant malleolar fractures. Subsequently the authors developed the renowned Ottawa Ankle Rules. After implementation of this clinical decision rule, a relative reduction (RR) of 28% of ankle radiography was recorded in the intervention hospital, whilst the control site showed a 2% increase. A reduction of time spent in the Emergency Department (116 minutes vs. 80 minutes) was also found without any missed fractures or patient discontent [6]. Bachmann et al. even reported a 30-40% reduction of unnecessary radiographs in their systematic review of all literature on the accuracy of the Ottawa Ankle Rules [7].

In 2003, Cevik et al. evaluated the value of physical findings to predict fractures in patients with acute wrist trauma [8]. They included fifty-five patients in their study and concluded that edema, pain on grip and supination, pain on active and passive motion and localized tenderness can be valuable to predict a fracture.

Recently, Calco-Lorenzo et al. analyzed the possibility of creating a clinical decision rule for the assessment of conventional X-rays in acute wrist trauma [9]. They employed 46 different anamnestic and examination variables and included 179 patients in their study. They concluded that an age equal or higher than thirty-five, edema in the dorsum of the wrist, limited supination or radial deviation as compared to the contralateral wrist and pain on performing the distal radioulnar drawer test, was predictive of the presence of a distal radius fracture. However, they advised "a broader study should be undertaken to assess the feasibility of introducing a clinical decision rule".

Therefore, the primary aim of this study is to formulate a clinical decision rule to determine when an X-ray of the distal radius is necessary in patients presenting to the Emergency Department following wrist trauma.

The second aim of this study is to validate this decision rule in a new patient population and to present it as simplified risk score.

## Methods/Design

This study consists of two components in which the data will be collected cross-sectionally. The data will be collected in three different hospitals during a period of six months. The participating hospitals include one university hospital, one non-academic teaching hospital, and one non-teaching hospital.

One component of this research comprises a study which will be performed to determine which combination of clinical parameters has a predictive value for the presence of a distal radius fracture in adult patients presenting to the Emergency Department following acute wrist trauma. This will be done by creating a model from the data collected in the university hospital and the non-academic teaching hospital. This model will be internally validated by bootstrapping. In the second component of this study, the developed prediction model will be validated in a new dataset. This dataset will be collected simultaneously in the third hospital, the non- teaching hospital, using the same approach as in the other hospitals.

### Study population

The study population is defined as all consecutive adult patients presenting at the Emergency Departments in the participating hospitals following wrist trauma and who are suspected to have sustained a distal radius fracture. Patients are suspected of having sustained a distal radius fracture when pain on pressure in the wrist area is indicated. The wrist is defined as the proximal segment of the hand consisting of the carpal bones and the associated soft parts and the distal segment of the ulnar and radial bone. A traumatic wrist injury is defined as a high or low energetic accident involving the wrist, e.g. a fall on the outstretched hand or a motor vehicle accident.

#### Inclusion criteria

• Patient aged 18 years and older

• An acute trauma of the wrist (< 72 hrs following trauma)

• Pain on pressure in wrist area

#### Exclusion criteria

• Pre-existent neurological pathology in the affected limb

• Previous fracture of distal radius <3 months

• Patients referred from another hospital where X-rays of the wrist were performed

• Multi trauma patient

• Patients who are not considered to be competent or capable by their treating physician to answer any questions regarding pain or mechanism of trauma as listed on the Case Report Form (Additional file 1).

#### Sample size

Due to the multivariable character of this study, a sample size calculation is not applicable, and we will therefore use a convenience sample. A common guideline is that the number of potential determinants should not exceed 10% of the number of events, in this case distal radius fractures. The Case Report Form (CRF, additional file 1) lists 20 parameters, therefore requiring a minimum of 200 patients with a distal radius fracture (see appendix). In preparation of this study, we recently conducted a retrospective study in the participating hospitals to determine the number of X-rays performed for wrist trauma. In the academic hospital, it was found that 550 radiographs in patients with acute wrist trauma were performed during a one year period. In the non-academic teaching hospital, this figure was even higher: 2300 in one year. The percentage of radiographs which reveal a distal radius fracture is currently being determined in a separate study performed at these hospitals and is estimated to be about 50%. We expect that a period of six months will suffice to include enough patients (500) to formulate the decision rule. No strict guidelines for the required sample size of external validation studies exist. However, we aim to perform this analysis in a dataset which is of equal size as the development data set. Therefore, to validate the decision rule in a new population, this data will be collected simultaneously in the third hospital, the non-teaching hospital, using the same approach as in the other two hospitals.

## Outcome measurements

### Main study parameter/endpoint

The main study endpoint is a distal radius fracture as assessed by a blinded and independent skeletal radiologist on conventional X-rays series; one posterior anterior (PA) and one lateral view with the elbow in 24 degrees of flexion. A fracture is defined as the presence or disruption of one or more of the cortices of the distal radius. Malalignment in the distal radial ulnar joint and radial carpal joint is regarded as a potential fracture. Small avulsions at bony attachments sides of ligaments are considered to be a fracture as well.

### Study procedures

Data collection will take place from February 2011 till August 2011. During this period, patients presenting following an acute traumatic injury of the wrist will receive care as usual. The difference is that, upon presentation with wrist pain following trauma, an additional Case Report Form (CRF) containing multiple clinical parameters will be completed by the treating physician. These clinical parameters are defined by trauma-mechanism, physical examination and functional testing and include; mechanism of trauma; swelling of the distal radius, swelling of the Anatomical Snuffbox; visible deformity of the wrist; palpability of radial artery pulsations; pain on palpation of the distal radius, distal ulna, Anatomical Snuffbox, radial styloid, ulnar syloid and Lister's tubercle; pain on dorsal flexion, palmar flexion, supination, pronation ulnar deviation, radial deviation; difference in prehensile strength test between affected and unaffected side; outcome distal radial drawer test and pain on axial compression (see appendix). Radiographs of the distal radius will be performed according to Dutch guidelines; at least one posterior anterior (PA) and one lateral view with the elbow in 24 degrees of flexion. Additional imaging for injuries associated with wrist trauma, e.g. suspected fracture of the scaphoid, can be performed selectively and will be on account of the treating physician. The independent radiologist assessing the outcome using the reference standard (X-rays) will be blinded from the results of the CRF to prevent incorporation bias. The dichotomous nature (yes/no) of the CRF and the fact that it is a standardized way of history taking and examination, will decrease interobserver variability and thus minimize underestimation of the potential diagnostic value of the parameters.

Upon completion of the data collection in all three participating medical centers, a prediction rule will be developed based on data from two of the participating hospitals; the academic hospital and the non-academic teaching hospital. Since prediction rules always perform perfectly in the patient population they were generated from, a validation study in a new study population is required to assess its predictive quality. To ensure the most stringent form of validation, the rule will be validated in data from the third hospital, the non-teaching hospital. This method is also known as domain validation. The method of data collection in the third hospital will be similar and done simultaneously. This dataset will be comparable to the first regarding size and informative parameters and the model will be tested to verify its predictive value of the primary outcome: distal radius fracture. If necessary, the model will be updated and finally it will be presented as a simplified risk score.

#### Withdrawal of individual subjects

Because this is an observational cohort study, no informed consent will be obtained and therefore withdrawal is unlikely to occur. The treating physician can decide to withdraw a subject from the study for urgent medical reasons.

### Statistical analysis

The data from the academic hospital and the non-academic teaching hospital will be used to develop the model. The data will be assessed by using a multivariable analysis. However, to prevent too optimistic estimates of the accuracy of the diagnostic model, a more liberal p-value for statistical significance will be employed (p = 0.15). Logistic regression modeling will be used to estimate the regression coefficients and thus the log odds of a fracture versus no fracture after which a reduced model will be created by means of a log likelihood ratio test.

The accuracy of the model will be estimated by a goodness of fit test and a ROC curve. If applicable, different models can be compared using the tests mentioned above. The final model will be validated internally through bootstrapping to estimate overfitting in the calibration and discrimination stated above. By averaging the optimism an adjusted model will be generated.

Henceforth, the prediction rule will be validated in a new data set, which will be collected simultaneously in the third hospital; the non-teaching hospital. If necessary, the model will be updated using the data from the validation study, by adjusting the intercept.

Finally, the prediction model will be presented as a simplified risk score which can be used by physicians to determine the need for an X-ray in patients presenting with wrist trauma at the Emergency Department.

## Ethical considerations

### Regulation statement

The study will be conducted according to the principles of the Declaration of Helsinki (59th World Medical Association General Assembly, Seoul, October 2008) and in accordance with the Medical Research Involving Human Subjects Act (WMO).

### Recruitment and consent

Patients with acute traumatic wrist injury will be treated by the physician on call in the Emergency Department. The only difference is that clinical parameters, as recorded during the physical examination will be more extensive and will be recorded more precise by the treating physician. Because of the observational character of this study, no informed consent was deemed necessary by the Medical Ethical Review Committee of the Academic Medical Center of the University of Amsterdam. This committee stated on March 17, 2011 that The Medical Research Involving Human Act (WMO) does not apply to this study and that an official approval by the committee is not required, reference number; project 2011_078.

## Administrative aspects and publication

### Handling and storage of data and documents

The data will be coded by patient number. Research data will be stored in a database (PASW statistics 18 and Microsoft Excel), and will be handled confidentially and anonymously. Research data that can be traced to individual persons can only be viewed by authorized personnel. These persons are the members of the research team, members of the health care inspection, and members of the Medical Ethics Committee of the Academic Medical Center Amsterdam. Review of the data may be necessary to ensure the reliability and quality of the research. The handling of personal data is in compliance with the Dutch Data Protection Act (in Dutch: 'Wet Bescherming Persoonsgegevens', WBP) and the privacy regulation of the Academic Medical Center Amsterdam.

## Discussion

As stated above, wrist trauma is frequently encountered at the Emergency Department. However, to this date, no decision rule regarding this type of trauma has been created.

Ideally, we would like to obtain radiographs of every patient entering one of the participating hospitals with trauma to the wrist. This however, is ethically and logistically not feasible. Therefore we should acquiesce to the fact that we obtain the informative parameters listed on the Case Report Form of each patient presenting with wrist pain post injury, but only outcome data on patients sent for X-ray by their treating physician. Any comments on this approach can be invalidated by stating that patients, for whom no radiography is required according to their physician, are not suspected of having a distal radius fracture and thus are not part of our domain.

## Competing interests

The authors declare that they have no competing interests. No external funding was received for this study.

## Authors' contributions

All authors participated in the design of the study and the drafting of the manuscript. All authors have read and approved the final manuscript.

## Pre-publication history

The pre-publication history for this paper can be accessed here:

http://www.biomedcentral.com/1471-2474/12/238/prepub

## Supplementary Material

Additional file 1 **Case Report Form**. the case report form containing the variables which will be filled out by the treating physician. Click here for file
