# Correction: Amsterdam wrist rules: a clinical decision aid

**DOI:** 10.1186/1471-2474-14-95

**Published:** 2013-04-02

**Authors:** Abdelali Bentohami, Monique MJ Walenkamp, Annelie Slaar, M Suzan H Beerekamp, Joris AH de Groot, Eva M Verhoog, L Cara Jager, Mario Maas, Taco S Bijlsma, Bart A van Dijkman, Niels WL Schep, J C Goslings

**Affiliations:** 1 Trauma Unit, Department of Surgery, Academic Medical Center, University of Amsterdam, P.O. Box 22660, Amsterdam, 1100 DD, The Netherlands; 2 Department of Clinical Epidemiology, University Medical Center, Utrecht, Huispost Str. 6.131, P.O. BOX 85500, Utrecht, 3508 GA, The Netherlands; 3 Department of Emergency Medicine, Academic Medical Center, University of Amsterdam, P.O. Box 22660, Amsterdam, 1100 DD, The Netherlands; 4 Department of Radiology, Academic Medical Center, University of Amsterdam, P.O. Box 22660, Amsterdam, 1100 DD, The Netherlands; 5 Department of Surgery-Traumatology, Spaarne Hospital, Spaarnepoort 1, Hoofddorp, 2134 TM, The Netherlands; 6 Department of Surgery-Traumatology, Flevo Hospital, Hospitaalweg 1, Almere, 1315 RA, The Netherlands

## 

The name of one of the authors of this manuscript [1] was misspelled. The correct name is: J. Carel Goslings. We regret any inconvenience this error has caused.

### Competing interests

All authors have no competing interests.

### Authors’ contribution

All authors have read and approved the final manuscript.

## Pre-publication history

The pre-publication history for this paper can be accessed here:

http://www.biomedcentral.com/1471-2474/14/95/prepub
