# Supplementary material for: Amsterdam wrist rules: A clinical decision aid
Source: BMC Musculoskelet Disord. 2011 Oct 17;12:238. doi: 10.1186/1471-2474-12-238 (PMC3229467; doi:10.1186/1471-2474-12-238)
Supplement: Additional file 1 — Case Report Form. the case report form containing the variables which will be filled out by the treating physician. [file 1471-2474-12-238-S1.DOC]

#
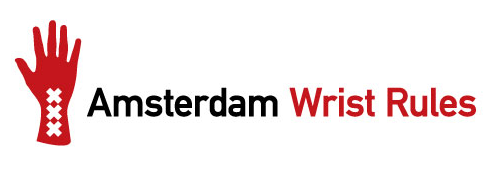

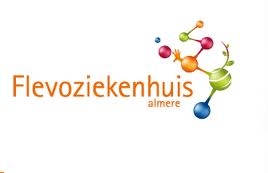

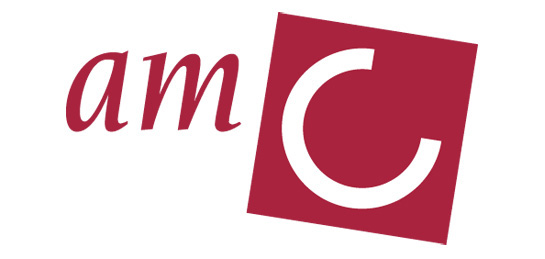


#
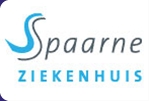


**Date: |__|__| - |__|__| - 2011**

**Patient information:**

Name:

Initials:

Date of Birth: |__|__| - |__|__| - |__|__|__|__|

Patient ID: |__|__|__|__|__|__|__|__|__|__|

Gender: □ male □ female

**Date trauma: |__|__| - |__|__| - 2011**

**Time trauma ………………………………**

**Name physician ………………………………..**

### Function: □ Emergency Medicine

### □ Surgery

### □ Orthopedics

### □ Other,…………………

**Affected side:** □ Left □ Right

**Dominant hand**: □ Left □ Right

## **Mechanism of Trauma**

- FOOSH (fall on outstretched hand)
- Motor Vehicle Accident
- Sports
- Other …………………

**Please circle**

| INSPECTION | | |
| --- | --- | --- |
| Swelling distal radius | Yes | No |
| Swelling Anatomical Snuffbox | Yes | No |
| Deformity | Yes | No |

| **ACTIVE MOVEMENT** | | |
| --- | --- | --- |
| Pain on dorsal flexion | Yes | No |
| Pain on palmar flexion | Yes | No |
| Pain on supination | Yes | No |
| Pain on pronation | Yes | No |
| Pain on ulnar deviation | Yes | No |
| Pain on radial deviation | Yes | No |

| **PALPATION** | | |
| --- | --- | --- |
| Radial artery pulsations palpable? | Yes | No |
|

| **Prehensile strength** |  | | |
| --- | --- | --- | --- |
| Measurement # | 1 | 2 | 3 |
| Unaffected side |  |  |  |
| Affected side |  |  |  |

| **Painful at palpation?** | | |
| --- | --- | --- |
| Distal radius | Yes | No |
| Distal ulna | Yes | No |
| Anatomical Snuffbox | Yes | No |
| Radial Styloid | Yes | No |
| Ulnar Styloid | Yes | No |
| Lister’s Tubercle | Yes | No |

| **FUNCTIONAL TESTS** | | |
| --- | --- | --- |
| Positive distal radioulnar drawer test? | Yes | No |
| Pain on axial compression? | Yes | No |

| Please indicate on this VAS scale your estimated probability of a distal radius fracture.  **0|--------------------------------------------------------------------------------------------------------| 100%** |
| --- |

**Please deposit this form in the mail box.**

**Thank you very much!**

##### Illustration of tests


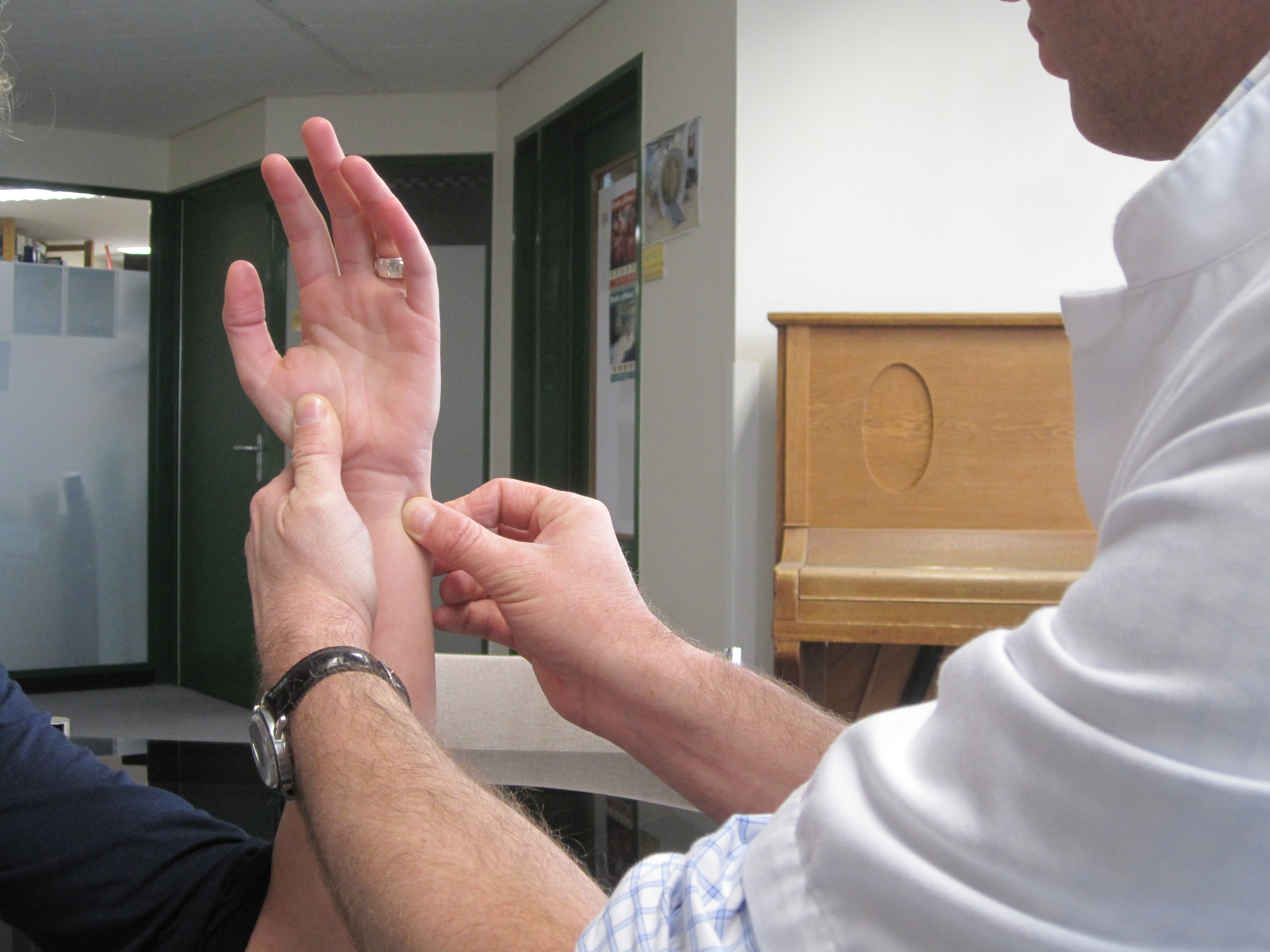

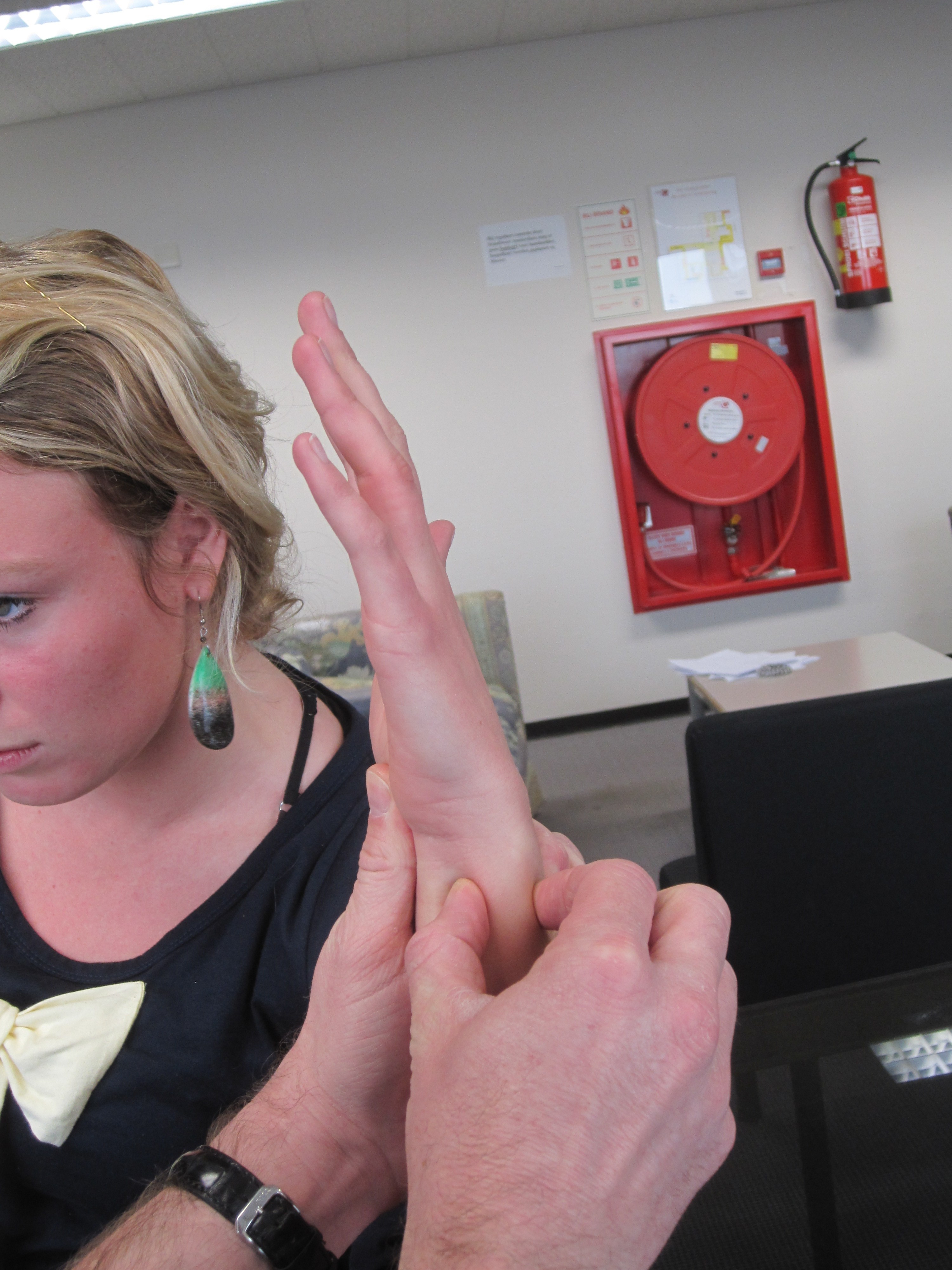
**Distal radioulnar drawer test**


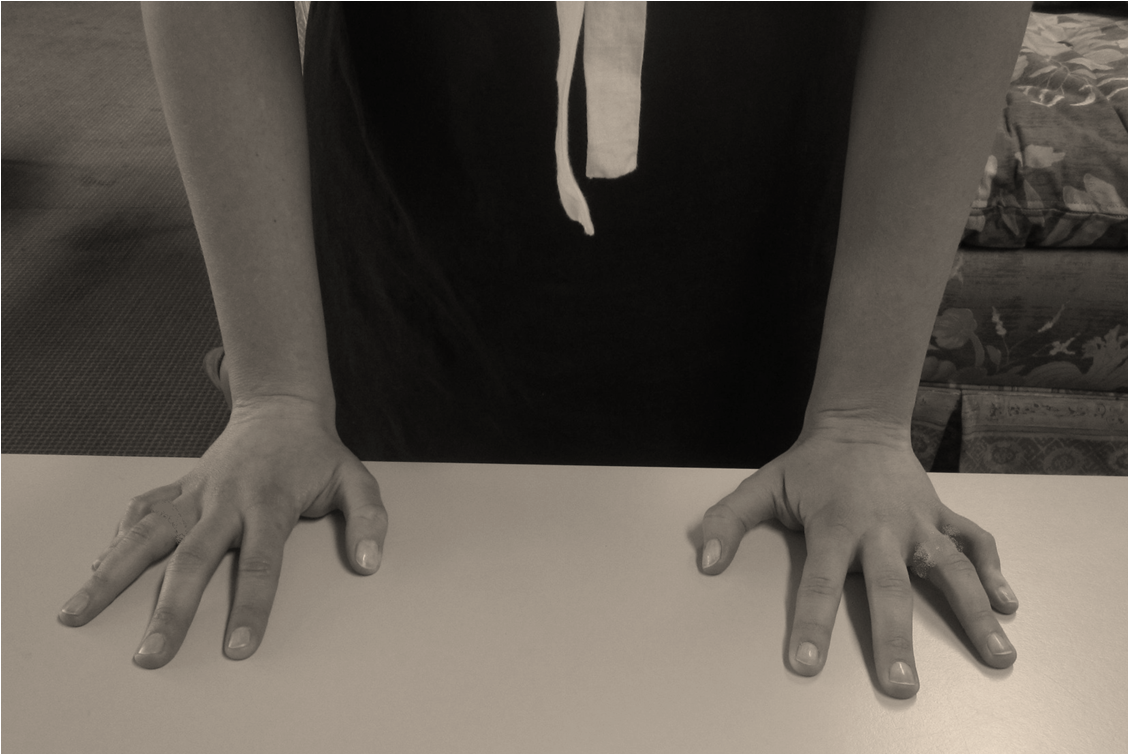
**Axial compression**


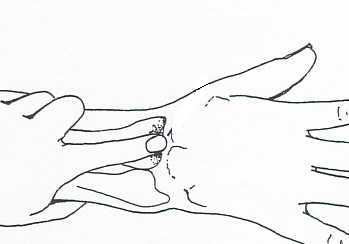
**Lister’s Tubercle**
